# Complete Genome Sequence of *Caulobacter crescentus* Siphophage Seuss 

**DOI:** 10.1128/genomeA.01132-15

**Published:** 2015-10-01

**Authors:** Jordan M. Sloan, Jennifer L. Keene, Jesse L. Cahill, Eric S. Rasche, Gabriel F. Kuty Everett

**Affiliations:** Center for Phage Technology, Texas A&M University, College Station, Texas, USA

## Abstract

*Caulobacter crescentus* is a water-dwelling bacterium known to have a dimorphic life cycle. Here, we announce the complete genome of Seuss, a *C. crescentus* icosahedral siphophage, and describe key features. Seuss is unique among phages deposited in GenBank, with genes encoding novel hypothetical proteins composing 45% of its genome.

## GENOME ANNOUNCEMENT

*Caulobacter crescentus* is a Gram-negative alphaproteobacterium found in nutrient-poor aquatic environments. It has a dimorphic life cycle and has been used to study cell differentiation and asymmetric division (1). Bacteriophages against *C. crescentus* have aided in the study of this model organism (2). Phages previously deposited in GenBank for this bacterium include myophage Cr30 and prolate-siphophage phiCbk and its relatives (3, 4). Here, we describe phage Seuss, a novel icosahedral siphophage infecting *C. crescentus*.

Bacteriophage Seuss was isolated from a water sample collected in San Antonio, TX, based on its ability to grow on *C. crescentus* strain CB15. Phage DNA was sequenced using 454 pyrosequencing at the Emory GRA Genome Center (Emory University, GA, USA). Trimmed FLX Titanium reads were assembled into a single contig at 30.2-fold coverage using the Newbler assembler, version 2.5.3 (454 Life Sciences), at default settings. The contig was confirmed to be complete by PCR using primers that face the upstream and downstream ends of the contig. Products from the PCR amplification of the junctions of concatemeric molecules were sequenced by Sanger sequencing (Eton Bioscience, San Diego, CA). Genes were predicted using GeneMarkS (5) and corrected using software tools available on the Center for Phage Technology (CPT) Galaxy instance (https://cpt.tamu.edu/galaxy-public/). Morphology was determined using transmission electron microscopy performed at the Texas A&M University Microscopy and Imaging Center.

Suess has an 85,138-bp genome that has a coding density of 95.6%. The GC content is 61.4%, which is lower than that of its host at 67.2% (1). Seuss contains 120 predicted coding sequences, 33 of which have putative functions based on BLASTp and InterPro Scan analyses (6, 7). A 1,748-bp terminal repeat was predicted using the PAUSE method (https://cpt.tamu.edu/pause/) on raw sequencing data. BLASTn analysis shows that Seuss has no relatives in the current database and is a novel phage (8).

Genes related to DNA replication and recombination were identified, including a primase, ligase, DNA polymerase, nucleases, and a Holliday junction resolvase. Few genes encoding morphogenesis and DNA packaging proteins were annotated, including tape measure protein, a minor tail protein, a tail fiber protein, the capsid protein, and the large terminase. A tape measure chaperone and conserved translational frameshift product were annotated as well (9). Additional morphogenesis and DNA packaging proteins could not be identified due to a lack of sequence homology and conserved domains. Biosynthesis genes were found, including ribonucleotide reductase small and large subunits, thymidylate synthase, and nucleoside hydrolases. Interestingly, a 1,680-bp region of the gene encoding the large ribonucleotide reductase subunit shows 79% nucleotide sequence identity to a region on the CB15 chromosome, suggesting a possible bacterial origin of the protein. To accomplish lysis, Seuss encodes a lysis cassette that consists of a putative holin/antiholin pair, soluble endolysin, and inner- and outer-membrane spanins. Additionally, *rIIa* and *rIIb* homologs were identified.

### Nucleotide sequence accession number.

The genome sequence of phage Seuss was contributed as accession number KT001914 to GenBank.
